# Characteristics of adverse drug reactions and risk management strategies for methylprednisolone sodium succinate in the treatment of idiopathic sudden sensorineural hearing loss: a clinical study of 1026 patients

**DOI:** 10.3389/fmed.2025.1589531

**Published:** 2025-06-11

**Authors:** Ting Wu, Yu Tang, Sinan Yan, Jie Meng, Shuangba He, Wei Meng

**Affiliations:** Department of Otorhinolaryngology Head and Neck Surgery, Nanjing Tongren Hospital, School of Medicine, Southeast University, Nanjing, China

**Keywords:** methylprednisolone sodium succinate, ISSNHL, ADRs, evidence-based medicine, clinical management

## Abstract

**Objective:**

To systematically analyze the patterns, risk factors, and clinical management strategies of adverse drug reactions (ADRs) associated with methylprednisolone sodium succinate in the treatment of idiopathic sudden sensorineural hearing loss (ISSNHL).

**Methods:**

A retrospective study was conducted on 1026 ISSNHL patients treated with methylprednisolone sodium succinate at Nanjing Tongren Hospital from January 2020 to December 2024. The incidence, types, severity, and outcomes of ADRs were recorded. Multivariate logistic regression was used to identify risk factors, and evidence-based intervention recommendations were proposed.

**Results:**

The overall ADRs incidence was 17.2% (177/1026), with gastrointestinal reactions (43.5%), elevated blood glucose (29.9%), and neuropsychiatric symptoms (18.1%) being the most common. Advanced age (≥60 years, OR = 2.24) and diabetes (OR = 3.15) were independent risk factors for ADRs (both *P* < 0.05). Symptoms were alleviated in 98.3% of patients after intervention, and treatment was discontinued in 3 cases due to severe hyperglycemia.

**Conclusion:**

The risk of ADRs from methylprednisolone sodium succinate is closely related to patients’ underlying conditions and treatment regimens. A stratified warning system and optimized individualized treatment strategies are necessary.

## 1 Introduction

Idiopathic sudden sensorineural hearing loss (ISSNHL) (1) is an otolaryngological emergency with an incidence of approximately 5 to 20 per 100,000 (2). Its pathological mechanisms involve inner ear microcirculation disorders, viral infections, and immune-inflammatory responses (3). Due to their potent anti-inflammatory effects, glucocorticoids are recommended as one of the first-line treatments in both domestic and international guidelines (4). Methylprednisolone sodium succinate, with its long half-life and low risk of water and sodium retention, is widely used (5). However, systemic adverse reactions to glucocorticoids (such as gastrointestinal ulcers, blood glucose fluctuations, and psychiatric abnormalities) may offset their therapeutic benefits, especially in patients with metabolic diseases or the elderly (6, 7). Most current studies focus on the hearing recovery rate in ISSNHL (1, 8), with few large-sample studies on ADRs associated with glucocorticoid treatment for ISSNHL, and a lack of risk stratification evidence for the Chinese population. This study aims to reveal the characteristics and management strategies of ADRs associated with methylprednisolone sodium succinate by analyzing clinical data from 1026 patients, providing a basis for safe clinical use.

## 2 Materials and methods

### 2.1 Study subjects

This single-center, retrospective cohort study included 1026 ISSNHL patients treated with methylprednisolone sodium succinate at Nanjing Tongren Hospital from January 2020 to December 2024. Inclusion criteria: Diagnosis consistent with the “Diagnosis and Treatment Guidelines for Sudden Deafness (2019)”; ① Received intravenous methylprednisolone sodium succinate for ≥3 days; ② Age ≥ 18 years. Exclusion criteria: ① Pregnant or lactating women; ② History of glucocorticoid allergy; ③ Severe liver or kidney dysfunction; ④ Use of glucocorticoids within one week before hospitalization; ⑤ Patients with missing data (27 individuals who have not completed the follow-up visits).

### 2.2 Treatment regimen

Initial dose: 60–80 mg/day intravenous infusion, reduced to 40 mg/day after 3 days, with a total treatment duration of 7–10 days (2).

Combined medication: All patients received Ginkgo biloba extract (Ginaton^®^) and mecobalamin to improve microcirculation.

### 2.3 Data collection and processing

Data sources: Hospital electronic medical record system (HIS) and pharmacy prescription database. Collected variables: ① Demographic characteristics (age, gender, BMI); ② Underlying diseases (diabetes, hypertension, history of gastric ulcers); ③ Treatment regimen (dose, duration, combined medications); ④ ADRs events (type, time of occurrence, severity, intervention measures).

### 2.4 ADRs assessment and grading

Definition: According to the WHO-UMC causality assessment criteria (9), a Naranjo score > 4 was considered “probable”or “definite” related. Grading criteria: Mild: Mild symptoms, no need to discontinue treatment; Moderate: Requires symptomatic treatment or dose adjustment; Severe: Life-threatening or causing permanent damage, requires emergency intervention. In our study, the determination of elevated blood glucose is as follows: the absolute increase in fasting blood glucose compared to the baseline is ≥1.7 mmol/L or the relative increase is ≥20%. Blood pressure fluctuation: the increase in blood pressure compared to the baseline is ≥20 mmHg (SBP) or ≥10 mmHg (DBP). Insomnia assessment criteria: a score of ≥15 points on the insomnia severity index indicates clinically significant insomnia. An increase of ≥7 points after medication compared to the baseline suggests drug-related insomnia. Anxiety assessment criteria: a score of ≥10 points on the Generalized Anxiety Disorder Scale-7 indicates an anxiety state. An increase of ≥4 points after medication compared to the baseline has clinical significance.

### 2.5 Statistical analysis

Data analysis was performed using SPSS 26.0. Measurement data were analyzed using *t*-tests, count data were analyzed using χ^2^ tests, and multivariate analysis was conducted using logistic regression models (*P* < 0.05)was considered statistically significant). All continuous variables were evaluated for normality using the Shapiro-Wilk test (α = 0.05).

## 3 Results

### 3.1 ADRs incidence and types

Overall incidence: 177 cases (17.2%) experienced ADRs, including 143 mild cases (80.8%), 29 moderate cases (16.4%), and 5 severe cases (2.8%). The distribution of types is as follows (Table 1).

**TABLE 1:** The ADRs incidence and types.

The category of ADR	(*n* = 177)	Percentage(%)
Gastrointestinal reactions	77	43.5
Elevated blood glucose	53	29.9
insomnia/anxiety	32	18.1
blood pressure fluctuations	10	5.6
rash	5	2.8

### 3.2 ADRs risk factor analysis

Univariate analysis indicated that the occurrence of adverse reactions in idiopathic sudden deafness patients treated with methylprednisolone sodium succinate was not statistically significant in relation to gender, BMI, allergy history, and hypertension history (*P* > 0.05), while there were significant differences when compared with age and diabetes history (*P* < 0.05) (Table 2). A multivariate Logistic regression analysis was conducted using the independent variables with statistical significance in the univariate analysis as the dependent variables. The results indicated that age ≥ 60 years (OR = 2.24, 95% CI = 1.45–3.47) and history of diabetes (OR = 3.15, 95% CI = 1.98–5.02) were high-risk factors for the occurrence of adverse drug reactions in patients with idiopathic sudden deafness who used methylprednisolone succinate sodium (*P* < 0.05) (Table 3).

**TABLE 2:** Single-factor analysis of the factors influencing the occurrence of ADRs in patients with ISSNHL due to the use of methylprednisolone succinate sodium.

Variable	Condition	Number (*n* = 1026)	Number of ADR cases	The incidence rate of ADR (%)	X^2^	*P*
Age	<60	613	76	12.4	25.04	<0.001
	≥60	413	101	24.5		
Gender	Man	499	85	17.0	0.024	0.877
	Woman	527	92	17.5		
BMI	<24	616	99	16.1	1.47	0.225
	≥24	410	78	19.0		
Allergy	Yes	92	20	21.7	1.4	0.237
	No	934	157	16.8		
Hypertension	Yes	308	61	19.8	2.72	0.099
	No	718	116	16.1		
Diabetes	Yes	194	69	35.6	56.3	<0.001
	No	832	108	13.0		

**TABLE 3:** Multivariate logistic analysis of multiple factors influencing the occurrence of adverse drug reactions in patients with idiopathic sudden deafness due to the use of methylprednisolone sodium succinate.

Variable	β	S.E	Waldχ ^2^	*P*	*OR*	95%CI
Age ≥ 60	0.806	0.245	10.82	<0.001	2.24	1.45–3.47
Diabetes	1.147	0.236	23.61	<0.001	3.15	1.98–5.02

### 3.3 ADRs intervention and outcomes

Intervention measures: ① Gastrointestinal reactions: 77 cases were treated with proton pump inhibitors (omeprazole), and 12 cases were additionally treated with gastric mucosal protectants; ② Elevated blood glucose: 41 of 53 cases were managed with dietary adjustments and oral hypoglycemic agents, and 12 cases required insulin therapy. Outcomes: Symptoms were alleviated in 172 cases (97.2%), and treatment was discontinued in 5 severe ADRs cases (3 cases of hyperglycemic crisis, 2 cases of gastrointestinal bleeding), who were transferred to specialized treatment.

## 4 Discussion

Methylprednisolone sodium succinate, as a core treatment for idiopathic sudden sensorineural hearing loss (ISSNHL), has been widely recognized for its efficacy (10). However, the potential risks of adverse drug reactions (ADRs) may significantly affect treatment compliance and patient outcomes. This study systematically revealed the characteristics and risk factors of ADRs associated with methylprednisolone sodium succinate by analyzing clinical data from 1026 patients and provided targeted management recommendations for clinical practice.

In this study, gastrointestinal reactions were the most common (43.5%), and their mechanisms may be closely related to the multiple effects of glucocorticoids: ① Increased gastric acid secretion: Glucocorticoids inhibit phospholipase A2 activity, reducing prostaglandin E2 (PGE2) synthesis, which is a key mediator for gastric mucosal protection, furthermore, the lack of PGE2 leads to increased gastric acid secretion and thinning of the mucus layer, resulting in mucosal damage (11). ② Inhibition of mucosal repair: Glucocorticoids can downregulate epidermal growth factor (EGF) expression, delaying ulcer healing (12, 13). In this study, 12 patients experienced stomach pain with hidden blood positivity, and 2 cases progressed to gastrointestinal bleeding, indicating the need for enhanced gastric mucosal protection measures in high-risk populations (such as the elderly and those with a history of gastric ulcers). Meanwhile, Studies have shown that prophylactic use of PPIs can significantly reduce the risk of hormone-related gastric ulcers (14). Only 9.8% of high-risk patients in this study received prophylactic Proton Pump Inhibitors (PPIs), while 100% of those who experienced gastrointestinal reactions used PPIs, suggesting insufficient attention to gastric mucosal protection in clinical practice. Therefore, for patients with a history of gastric ulcer, prophylactic use of proton pump inhibitors (PPIs) can be considered to reduce gastrointestinal reactions.

Elevated blood glucose was the second most common ADRs (29.9%), showing a significant dose-dependent relationship. ① Enhanced gluconeogenesis: Glucocorticoids activate phosphoenolpyruvate carboxykinase (PEPCK) and glucose-6-phosphatase (G6Pase), promoting hepatic glucose output while inhibiting peripheral glucose uptake (15, 16). ② Insulin resistance: Glucocorticoids can reduce tyrosine phosphorylation of insulin receptor substrates (IRS), interfering with insulin signaling pathways (17). In this study, the risk of ADRs increased 3.15-fold in patients with diabetes (OR = 3.15), suggesting that such patients should be prioritized for low-dose regimens or local administration (such as intratympanic injection) (18).

Among the 53 patients with elevated blood glucose in this study, 12 (22.6%) required insulin therapy, and 3 cases experienced hyperglycemic crisis. It is recommended to monitor fasting and postprandial blood glucose daily in diabetic patients during treatment and at least every 3 days in non-diabetic patients. Furthermore, for high-risk patients (such as those aged ≥ 60 years and with diabetes), it is recommended to adopt an individualized treatment plan of “starting with 60 mg/d and reducing to 30 mg/d after 3 days”, which can ensure therapeutic efficacy while reducing cumulative exposure. For diabetic patients, regular monitoring of blood glucose levels is necessary, and dietary adjustments or the use of hypoglycemic drugs should be made when necessary. Moreover, for elderly patients, the dosage should be adjusted according to liver and kidney functions to reduce the risk of adverse reactions caused by abnormal drug metabolism. Through these preventive measures, the safety of treatment can be significantly improved.

18.1% of the patients suffered from insomnia or anxiety, and the mechanism which may be involved extensive influences of N-methyl-D-aspartate (NMDA) receptor regulation in the central nervous system (CNS) (19). Meanwhile, glucocorticoids can penetrate the blood-brain barrier, enhancing NMDA receptor activity, leading to neuronal overexcitation and causing anxiety and sleep disorders.

The risk of ADRs increased 2.24-fold in elderly patients (OR = 2.24), possibly due to the following factors: ① Pharmacokinetic changes: Decreased liver enzyme activity in elderly patients leads to reduced drug clearance and increased blood concentration (20). ② Comorbidities: In this study, 58.3% of patients aged ≥ 60 years had diabetes or hypertension, and the superposition of multiple pathological states may further amplify ADRs risks (21). ③ Combined medication patterns: All patients in this study received Ginkgo biloba extract, whose antioxidant effects may partially counteract the oxidative stress damage caused by hormones, but no significant impact on ADRs incidence was observed.

This study has the bias of a retrospective design, and some mild ADRs (such as transient insomnia) may not have been fully recorded, leading to an underestimation of the incidence rate. Meanwhile, the sample was sourced from a single hospital, which may not accurately represent the real situation in primary healthcare institutions. Additionally, the lack of long-term follow-up has not assessed the impact of ADRs on long-term metabolism (such as osteoporosis) or hearing prognosis. Furthermore, we lack a control group with sufficient clinical samples for comparison. Future research could explore the correlation between CYP3A4 (22), GR (glucocorticoid receptor) (23) gene polymorphisms and ADRs to provide a basis for precise medication. Furthermore, establishing a “hormone medication management team” in collaboration with endocrinology and psychiatry departments to implement comprehensive monitoring for high-risk patients is recommended.

## 5 Conclusion

Methylprednisolone sodium succinate is a core treatment drug for ISSNHL, but its ADRs risks cannot be overlooked. By identifying high-risk populations, optimizing dosage strategies, and enhancing dynamic monitoring, the safety of medication use can be significantly improved. It is recommended that clinical practices establish a closed-loop management process of “assessment-intervention-reassessment” and conduct multicenter studies to validate the universality of the stratification scheme.

## Data Availability

The raw data supporting the conclusions of this article will be made available by the authors, without undue reservation.
